# Is it possible to identify the inguinal nerves during hernioplasty? A systematic review of the literature and meta-analysis of cadaveric and surgical studies

**DOI:** 10.1007/s10029-018-1857-2

**Published:** 2018-12-20

**Authors:** R. Cirocchi, B. M. Henry, I. Mercurio, K. A. Tomaszewski, P. Palumbo, A. Stabile, M. Lancia, J. Randolph

**Affiliations:** 1 Department of Surgical and Biomedical Sciences, University of Perugia, Perugia, Italy; 2 Department of Anatomy, Jagiellonian University Medical College, 12 Kopernika Street, 31-034 Kraków, Poland; 3 Department of Surgical Sciences, The University of Rome “La Sapienza”, Rome, Italy; 4 Tift College of Education, Mercer University, Atlanta, GA USA

**Keywords:** Hernioplasty, Iliohypogastric nerve, Ilioinguinal nerve, Genital branch of the genitofemoral nerve, Iatrogenic injury

## Abstract

**Purpose:**

Patients who undergo inguinal hernioplasty may suffer from persistent postoperative pain due to inguinal nerve injuries. The aim of this systematic review and meta-analysis was to provide comprehensive data on the prevalence (identification rates), anatomical characteristics, and ethnic variations of the ilioinguinal (IIN), the iliohypogastric (IHN) and the genital branch of the genitofemoral (GNF) nerves.

**Methods:**

The systematic literature search was conducted using the PubMed, Scopus and Web of Science databases.

**Results:**

A total of 26 articles (5265 half-body examinations) were included in this study. The identification rate of the IIN was 94.4% (95% CI 89.5–97.9) using a random-effects model. Unweighted multiple regression analysis showed that study sample size (*β* = − 0.74, *p* = .036) was the only statistically significant predictor of lower prevalence. The identification rates of the IHN and GNF was 86.7% (95% CI 78.3%–93.3%) and 69.1% (95% CI 53.1%–83.0%) using a random-effects model, respectively. For those outcomes, a visual analysis of funnel and Doi plots indicated irregularity and provided evidence that larger studies tended to have lower identification rates. In terms of the synthesis of anatomical reference points, there was a large and statistically significant amount of heterogeneity for most outcomes.

**Conclusions:**

The identification rates of the inguinal nerves in our study were lower than reported in literature. The lowest was found for GNF, suggesting that this nerve was the most difficult to identify. Knowledge regarding the anatomy of the inguinal nerves can facilitate their proper identification and reduce the risk of iatrogenic injury and postoperative pain.

## Introduction

Knowledge of the course of nerves in the inguinal region is essential for the treatment of hernia. Proper nerve identification during open hernia surgery can influence the incidence of postoperative chronic pain [1].

The inguinal canal runs through the muscles of the abdominal wall in an oblique direction, downward and medially, allowing for the passage of the spermatic cord (male) and round ligament (female). It is bounded by the transverse fascia posteriorly, the aponeurosis of external oblique anteriorly, the inguinal ligament inferiorly, and the bottom edge of the internal oblique and transverse abdominal muscles superiorly [2]. The canal has two openings: the upper one (internal inguinal ring) and the lower one (external inguinal ring).

**Fig. 1:**
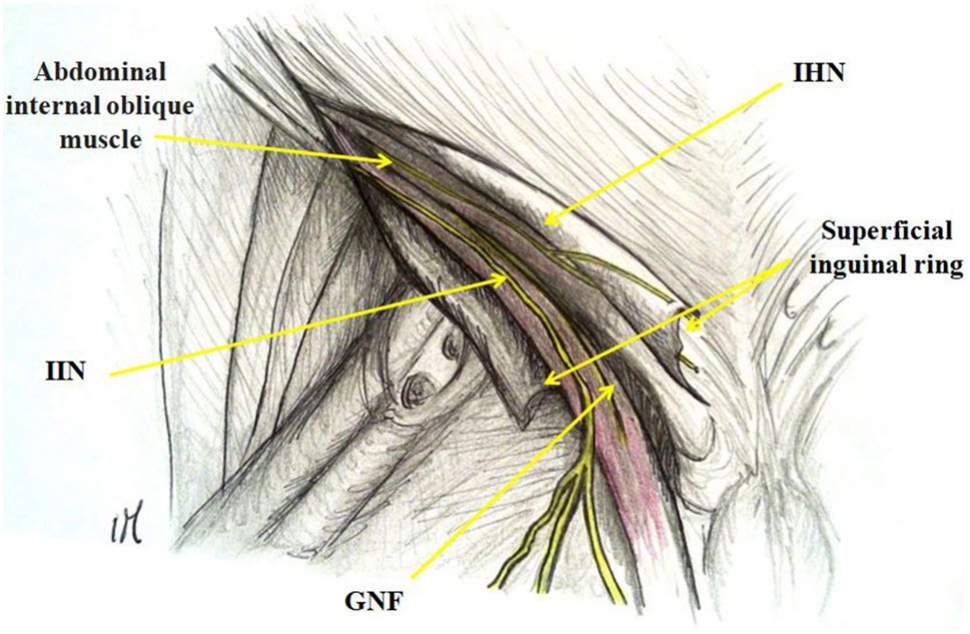
Anatomy of the inguinal region

The inguinal canal is also crossed by the iliohypogastric (IHN), ilioinguinal (IIN) and the genital branch of the genitofemoral nerves (GNF) (Fig. 1). These nerves are the terminal branches of the lumbar plexus and innervate the abdominal muscle and the skin of genitals, buttock, and hypogastric region [3]. The IHN, IIN, and GNF are potentially at risk of iatrogenic injury during common surgical procedures, such as caesarean section, inguinal hernioplasty and most laparoscopic procedures.

Anatomical variants of the nerves in the inguinal region have been reported in the literature, but their prevalence is heterogeneous across different studies [4]. Patients who undergo inguinal hernioplasty may suffer from persistent postoperative pain, with an incidence that varies from 0.7 to 43.3% and with a rate of debilitating pain that varies from 0.5 to 6% [5, 6]. Previous research showed that failure to identify inguinal nerves is correlated with the presence of chronic pain [7]. Moreover, the incidence of this complication increases with the number of undetected nerves [2]. Having detailed knowledge on the inguinal nerves can significantly improve the safety and success rate of several surgical procedures besides inguinal hernia repair, such as varicocele surgery and ilioinguinal/iliohypogastric blocks with ultrasound-guided or landmark-based techniques [8–10].

The current European Hernia Society guidelines suggest the identification of the three inguinal nerves to decrease late postoperative pain, but in clinical practice, the fundamental question is: “Is it possible to identify every inguinal nerve during hernioplasty?” [10]. The aim of this systematic review and meta-analysis on inguinal nerves was to analyze and provide comprehensive data on their prevalence (identification rates), anatomical characteristics, and possible sources of heterogeneity, to decrease the risk of iatrogenic injury to these nerves during inguinal surgery.

## Materials and methods

### Study selection

A systematic review was performed on studies assessing the anatomical variations of inguinal nerves in accordance with the Preferred Reporting Items for Systematic Reviews and Meta-analyses (PRISMA) standards. The systematic literature search was conducted using the PubMed, Scopus and Web of Science database engines employing the terms: “inguinal” and “nerve” or “ilioinguinal” and “nerve” or “iliohypogastric” and “nerve” or “genitofemoral” and “nerve”. No language or publication date restrictions were imposed.

Two authors (RC and MB) independently screened full-text papers for eligibility. When multiple articles were published from a single study group and when overlapping study periods were reported, only the most recent article was considered to avoid duplication of data. The PubMed function “related articles” was used to broaden each search and the reference list of all potentially eligible studies was analyzed. To minimize retrieval bias, a manual search including the Science Citation Index Expanded, Scopus and Google Scholar databases was performed. The final decision on eligibility was reached by consensus between the two screening authors.

### Inclusion and exclusion criteria

To be included in the present meta-analysis, a study had to report clear anatomical identification of inguinal nerves as primary or secondary outcomes in cadaveric or prospective operative studies. Case reports, editorials, conference abstracts, and studies reporting incomplete or irrelevant data were excluded.

A protocol for this meta-analysis was registered on PROSPERO: CRD42017074589 (http://www.crd.york.ac.uk/prospero).

### Data extraction

We developed a data extraction sheet based on the Cochrane Consumers and Communication Review Group’s data extraction template. Two authors (RC and MB) independently retrieved data from the included studies. A third author (JR) checked the extracted data. Disagreements were solved through discussion and, if necessary, by involving an independent fourth author (CR).

### Outcomes

The primary outcome of interest was the prevalence (identification rate) of the inguinal nerves: IIN, IHN, or GNF.

The following anatomical reference points were considered as secondary outcomes:

- Distance of the emergence of the IIN from abdominal wall:

- inferiorly to the anterior superior iliac spine,
- medially to the anterior superior iliac spine.

b. Variations in the emergence of the IIN posteriorly to:

- the inguinal ligament,
- the anterior superior iliac spine (ASIS).

c. Aberrant origin of the IIN from the GNF.

d. IIN common trunk with the IHN.

e. Course of the IIN with regard to the spermatic cord:

- parallel,
- ventral.

f. Type of exit of IIN from inguinal canal:

- IIN exit through superficial inguinal ring (SIR)
- Acute infero-lateral angulation of the IIN in close contact with and parallel to the SIR fibers at exit.
- A plane superficial to the external oblique aponeurosis (EOA) having pierced it proximal to the SIR.

g. Mode of termination and branches.

### Statistical analysis

Binomial pooled prevalence estimates (PPEs) (i.e., identification rates) for the IIN, IHN, GNF, and anatomical reference points were computed using MetaXL software (version 5.0). Other analyses were completed with SPSS 24.0. The *I*^2^ statistic and its 95% confidence interval and Cochrane’s *Q* and significance level were reported as indicators of heterogeneity. We examined funnel and DOI plots for outcomes with ten or more studies. Where there was significant asymmetry in those plots, we conducted a sensitivity analysis between a random-effects model and an inverse variance fixed-effects model with a heterogeneity correction [11, 12] as suggested in Sterne et al. [13]. In addition, we conducted a leave-one-out sensitivity analysis for overall outcomes with 10 or more studies. In one study, we estimated the standard deviation from the range using the recommendations in Hozo, Djulbegovic, and Hozo [12]. Subgroups analyses were conducted for type of dissection (cadaveric or during hernioplasty), geographical region (Africa, Asia, Europe, North America, or South America), and the number of study centers (single center or multicenter). We also examined year and study sample size as possible sources of heterogeneity. An unweighted multiple regression analysis was carried out to identify the degree to which each of the following predictors, in concert, were associated with the IIN identification rate: type of dissection, geographical region, number of study centers, year of publication and study sample size.

## Results

**Fig. 2:**
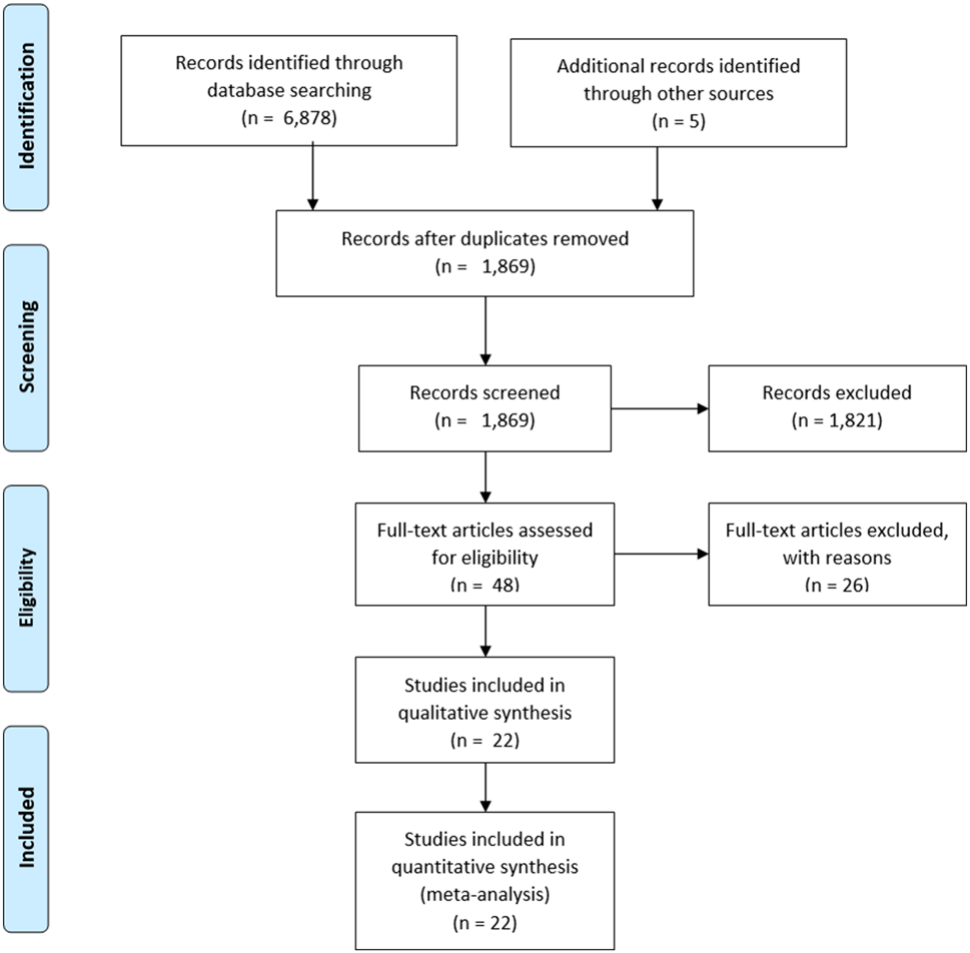
PRISMA flow diagram

The PRISMA flow diagram for the systematic review is presented in Fig. 2. The initial search yielded 6878 potentially relevant articles. After removing 5014 duplicates and assessing titles/abstracts for eligibility, 1821 further articles were eventually excluded. Forty-eight studies were analyzed in full-text. Of these, 22 were excluded because the primary outcome of our review was not described. Finally, 26 articles were included in this systematic review and meta-analysis (Table 1) [2, 3, 7, 14–36].

**Table 1:** Included studies

Author, year of publication	Country	Type of study	Single center/multicenter	*n* = (# half-bodies studied)
Mendes 2016 [14]	Brazil	Cadaveric	Single center	10
		During hernioplasty	Single center	29
Smeds 2016 [15]	UK/Sweden/The Netherlands	During hernioplasty	Multicenter	507
Grossi 2015 [16]	Brazil	During hernioplasty	Single center	38
Sanders 2014 [17]	UK/Sweden	During hernioplasty	Multicenter	553
Pandhare 2013 [3]	India	Cadaveric	Single center	40
Emeksiz 2013 [18]	Turkey	During hernioplasty	Single center	116
Yıldız 2012 [19]	Turkey	Cadaveric	Single center	34
Bischoff 2012 [20]	Denmark	During hernioplasty	Single center	244
Klaasen 2011 [21]	USA	Cadaveric	Single center	200
Ergül 2011 [22]	Turkey	During hernioplasty	Single center	25
Smeds 2010 [23]	Sweden	During hernioplasty	Single center	525
Ndiaye 2010 [24]	France	Cadaveric	Single center	100
Lange 2009 [25]	The Netherlands	During hernioplasty	Single center	40
Wijsmuller 2007 [2]	The Netherlands	Cadaveric	Single center	18
Bartlett 2007 [26]	UK	During hernioplasty	Single center	172
Mui 2006 [27]	China	During hernioplasty	Single center	100
Alfieri 2006 [7]	Italy	During hernioplasty	Multicenter	973
Picchio 2004 [28]	Italy	During hernioplasty	Single center	813
Ducic 2004 [29]	USA	Cadaveric	Single center	20
Al-dabbagh 2002 [30]	UK	During hernioplasty	Single center	110
Rab 2001 [31]	USA	Cadaveric	Multicenter	64
Diop 2000 [32]	Senegal	Cadaveric	Single center	40
Ravichandran 2000 [33]	UK	During hernioplasty	Single center	40
Mandelkow 1988 [34]	Germany	Cadaveric	Single center	88
Salama 1983 [35]	France	Cadaveric	Single center	25
Papadopoulos 1981 [36]	Greece	Cadaveric	Single center	341
Total				5265

Twenty-six included studies reported 5265 half-body examinations. Fourteen studies were performed during inguinal hernioplasty, 12 studies were performed during cadaveric dissections. *A study by* Mendes et al. [14] reported two different dissection types: Mendes2016a was for cadaveric dissection; Mendes 2016b was for during hernioplasty—which were counted as independent effect measures for the purposes of analysis. All studies were prospective in design.

Most studies were conducted in Europe (Table 2). Fifteen studies were performed in Europe, five in Asia, three in North America, two in South America (both studies from Brazil), and one in Africa. No studies were performed in Australasia (Table 3). The nerve identification rates at the inguinal canal were evaluated (Table 3).

**Table 2:** Overall geographic localization and type of inguinal dissection

Continents	Type of inguinal dissection					
	Cadaveric		Hernioplasty		Total	
	Number of studies	*n* = half-bodies (% within region)	Number of studies	*n* = half-bodies (% within region)	Number of studies	*n* = half-bodies (% of total)
Europe	5	572 (12.58%)	10	3977 (87.42%)	15	4549 (86.4%)
Asia	2	74 (23.5%)	3	241 (76,5%)	5	315 (6%)
South America	1	10 (13%)	2*	67 (87%)	3*	77 (1.5%)
North America	3	284 (100%)	0	0	3	284 (5.4%)
Africa	1	40 (100%)	0	0	1	40 (0.7%)
Total	12	980 (18.62% of total)	14	4285 (81.38% of total)	**26**	**5265**

*One article includes cadaveric specimen and hernia repair in the same study which were counted as separate effect sizes

**Table 3:** Pooled prevalence estimates (identification rates) of nerves in the inguinal canal: geographical location

	Africa		Asia		South America (Brazil)		North America		Europe	
	Half-bodies analyzed (% of total sample size)	Number of nerves identified	Half-bodies analyzed (% of total sample size)	Number of nerves identified	Half-bodies analyzed (% of total sample size)	Number of nerves identified	Half-bodies analyzed (% of total sample size)	Number of nerves identified	Half-bodies analyzed (% of total sample size)	Number of nerves identified
IHN	0	0	25 (7.93%)	25	77 (100%)	71	200 (70.42%)	200	3885 (85.4%)	2811
Prevalence	0%		100%		92.2%		100%		72.35%	
IIN	40 (100%)	37	215 (68.3%)	212	77 (100%)	72	284 (100%)	284	3157 (69.4%)	2587
Prevalence	92.5%		98.6%		93.5%		100%		81.9%	
GNF	0	0	125 (39.7%)	121	77 (100%)	55	20 (7.04%)	20	3132 (68.8%)	1392
Prevalence	0%		96.8%		71.42%		100%		44.4%	

*IIN* ilioinguinal nerve, *IHN* iliohypogastric nerve, *GNF* genital branch of the genitofemoral nerve

### Meta-analysis on the identification rate of the ilioinguinal nerve

**Fig. 3:**
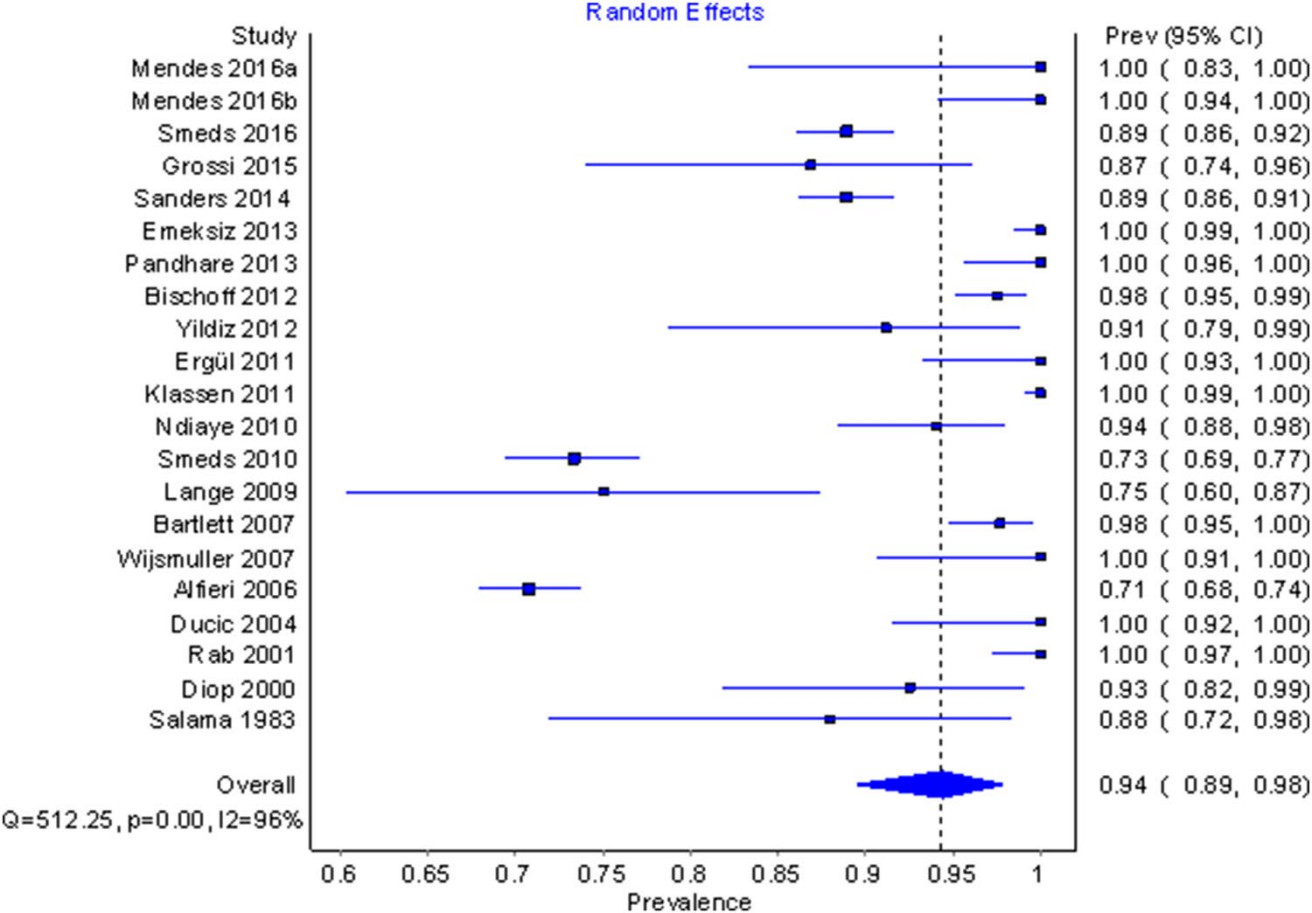
Pooled prevalence estimates (identification rates) of the IIN under a random-effects model

Figure 3 shows the identification rate of the IIN. A total of 21 studies and 3773 half-bodies were analyzed using a random-effects model (Table 4). The overall identification rate was 94.4% (95% CI 89.5–97.9). In a leave-one-out sensitivity analysis, the identification rates varied slightly from 93.7 to 95.2%. The funnel plot and DOI plot indicated major asymmetry; therefore, we also examined fixed-effect model with heterogeneity correction.

**Table 4:** Pooled prevalence estimates (identification rates) of the IIN

Study Group	*N*	Half-bodies	PPE% (95% CI) Random	PPE% (95% CI) Fixed	*I*^2^ (95% CI)	*Q*
All Studies	21	3773	94.4 (89.5–97.9)	87.0 (76.7–95.8)	96.1 (95.0–96.9)	512.2^***^
Type of dissection						
Cadaveric	10	551	97.3 (93.6–99.5)	98.1 (94.6–1.00)	70.6 (43.8–84.6)	30.6^***^
During hernioplasty	11	3222	91.4 (84.3–96.9)	84.3 (72.4–94.5)	97.1 (96.1, 97.9)	346.9^***^
Study center						
Single center	17	1676	95.9 (89.9–99.2)	92.3 (82.1–99.7)	94.2 (92.0–95.7)	274.4^***^
Multicenter	4	2097	90.1 (77.9–98.1)	82.0 (66.3–95.3)	97.9 (96.6–98.8)	146.2^***^
Geographic region						
Asia	1	40	91.8^a^ (81.9**–**99.0)	91.8^a^ (81.9**–**99.0)	NC	NC
Africa	1	40	99.4^a^ (95.7–1.00)	99.4^a^ (95.7–1.00)	NC	NC
Europe	13	3332	91.5 (84.9–96.5)	84.6 (73.5–94.2)	96.6 (95.4–97.5)	351.5^***^
North America	3	284	99.8^b^ (99.1–100.0)	99.8^b^ (99.1–100.0)	0.0 (0.0–57.9)	0.50
South America (Brazil)	2	77	95.2 (84.2–100.0)	94.7 (81.9–1.00)	67.3 (0.0–90.6)	6.13*

*PPE* pooled prevalence estimate, *NC* not computable because there was only one study in this group

*p < .05, **p < .01, ***p < .001

^a^Fixed- and random-effects estimates are identical because there was only one study in this subgroup

^b^Fixed- and random-effect estimates are identical because the study-level prevalence rates were all 100.0%

**Fig. 4:**
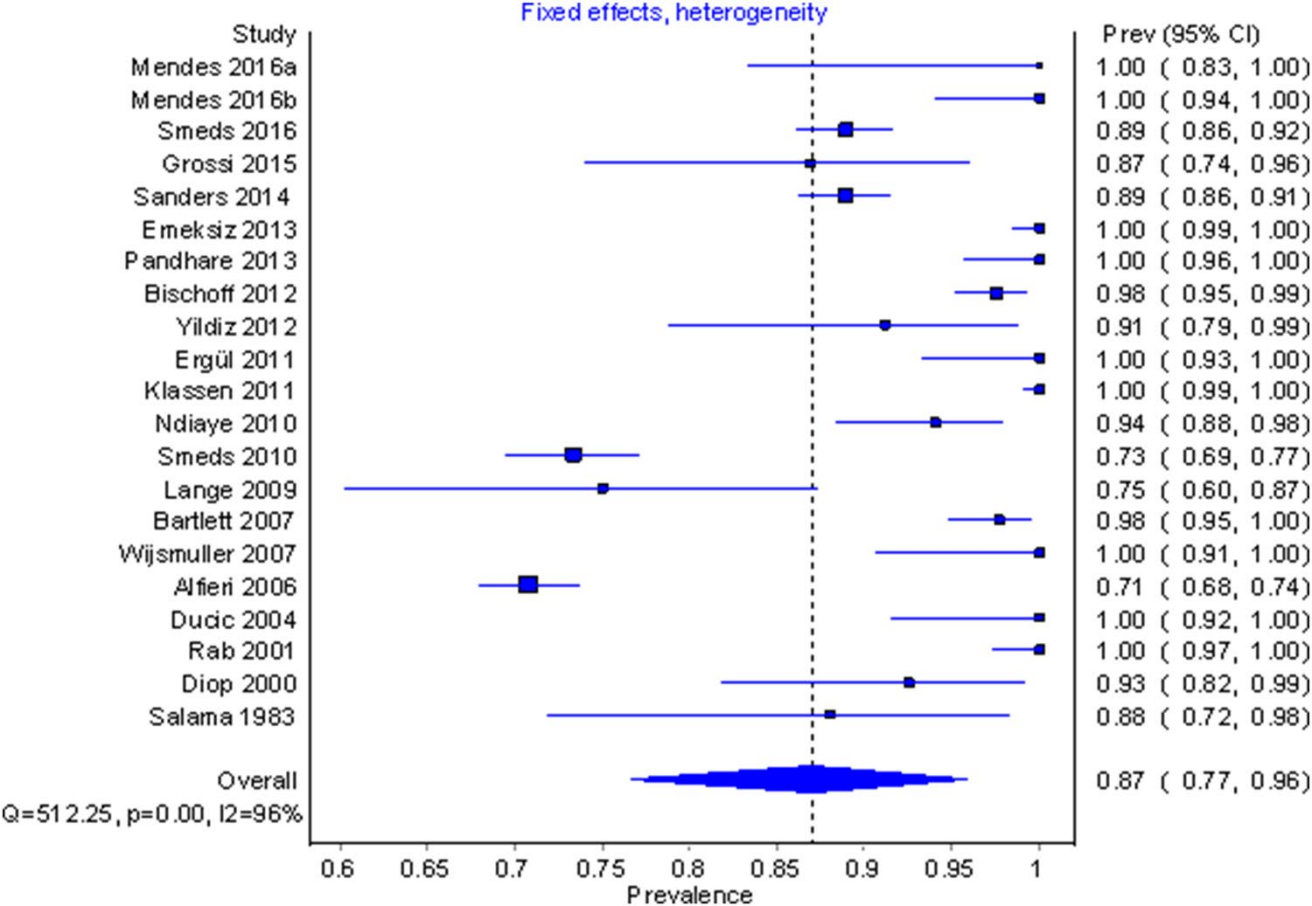
Pooled prevalence estimates (identification rates) of the IIN under a fixed-effects model with heterogeneity correction

**Fig. 5:**
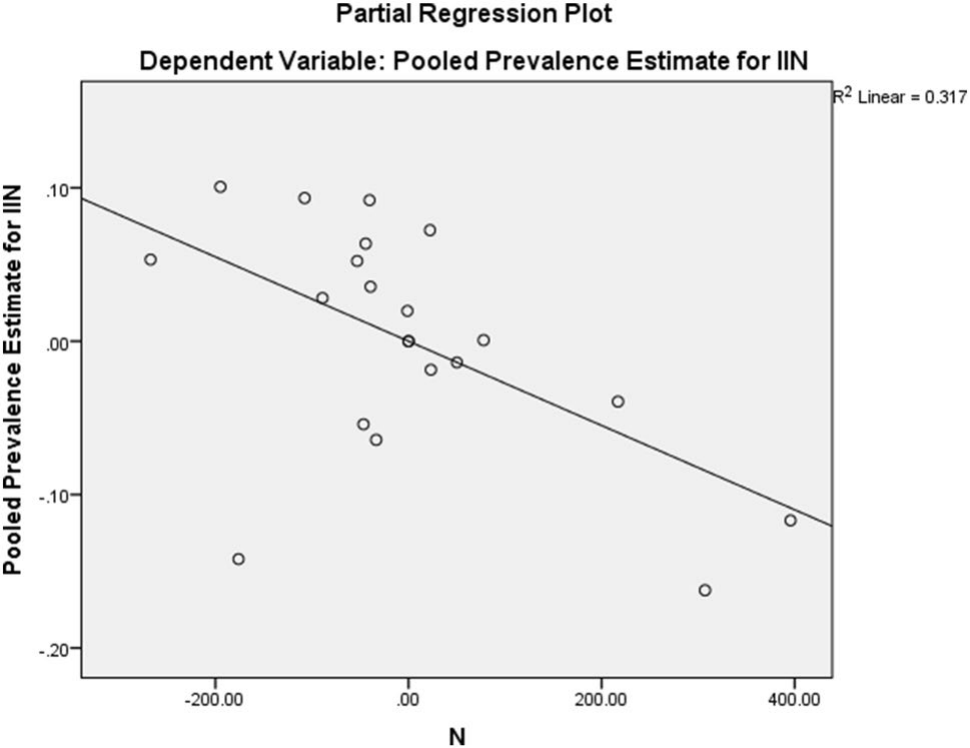
Partial regression plot of sample size and pooled prevalence estimates of IIN when controlling for region, type of dissection, and number of centers. Note that values are mean-centered at zero. The outlier in the bottom left corner is Lange (2014)—a small sample size study (*N* = 40) with a low prevalence estimate (75%)

Under the fixed-effect model with a heterogeneity correction, which gives more weight to large studies like Alfieri et al. [7] (*n* = 525) and Smeds et al. [15] (*n* = 973), the identification rate was 87.0% (95% CI 76.7%–95.8%) (Fig. 4). The median sample size for studies included in this analysis was 40. A follow-up unweighted multiple regression analysis showed that study sample size (*β* = − 0.74, *p* = .036) was the only statistically significant predictor for lower identification out of the following variables: sample size, year, region, number of centers, and type of dissection, (for the whole model: *R*^2^ = 0.56, *F*(8,12) = 1.94, *p* = .146). See Fig. 5 for a partial regression plot between sample size and PPE. The outlier in the bottom left of Fig. 5 was Lange et al. [25]—a study with a small sample size (*n* = 40) and a low identification rate (75.0%, 95% CI 60.3%–87.4%). For the remainder of this analysis, we assumed that the sample size/prevalence relationship was a source of bias and, therefore, we described results for both random-effect and fixed-effect models.

### Meta-analysis on the identification rate of the iliohypogastric nerve

**Fig. 6:**
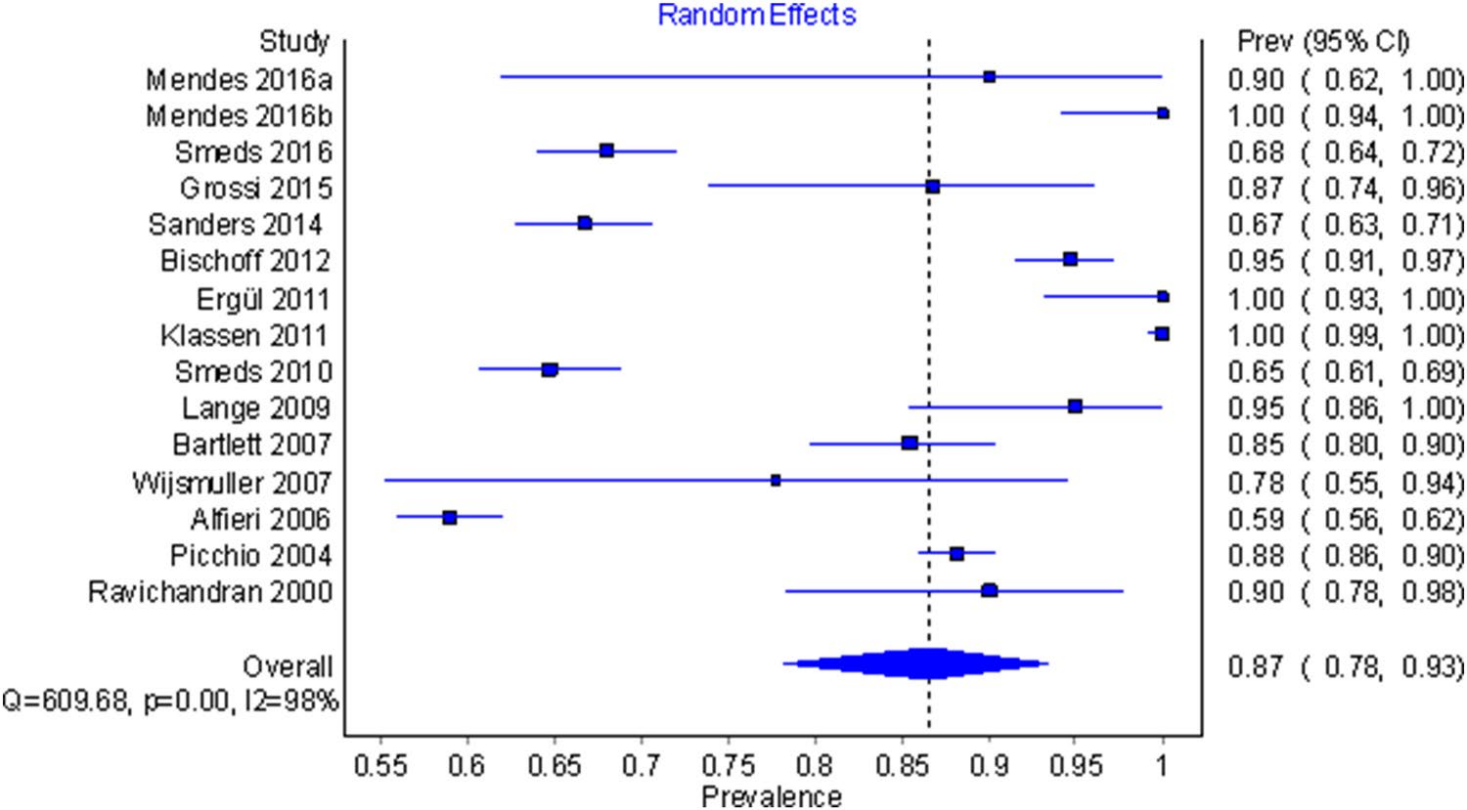
Pooled prevalence estimates (identification rates) of the IHN under a random-effects model

Figures 6 (random-effects model) and 7 (fixed-effects model) show the identification rate for IHN. A total of 15 studies and 4187 half-bodies were analyzed. The overall identification rate for the IHN was 86.7% (95% CI 78.3%–93.3%) and 76.3% (95% CI 62.5%–88.9%) using a random-effects model and fixed-effects model, respectively. In a leave-one-out sensitivity analysis, the identification rates varied slightly from 84.4 to 88.1% for a random-effects model and from 74.3 to 80.9% for a fixed-effects model. A visual analysis of a funnel plot and DOI plot indicated marked asymmetry; we also noted that there was a negative relationship between prevalence and sample size—similar to the IIN outcome. Table 5 shows the results of the overall and subgroup analyses for the IHN. The identification rates ranged from 64.5% (95% CI 58.5%–70.4%) for multicenter studies to 99.9% (95% CI 99.1%–100.0%) for single center studies. As in the IIN outcome, there was a large, statistically significant amount of heterogeneity overall and within subgroups.

**Fig. 7:**
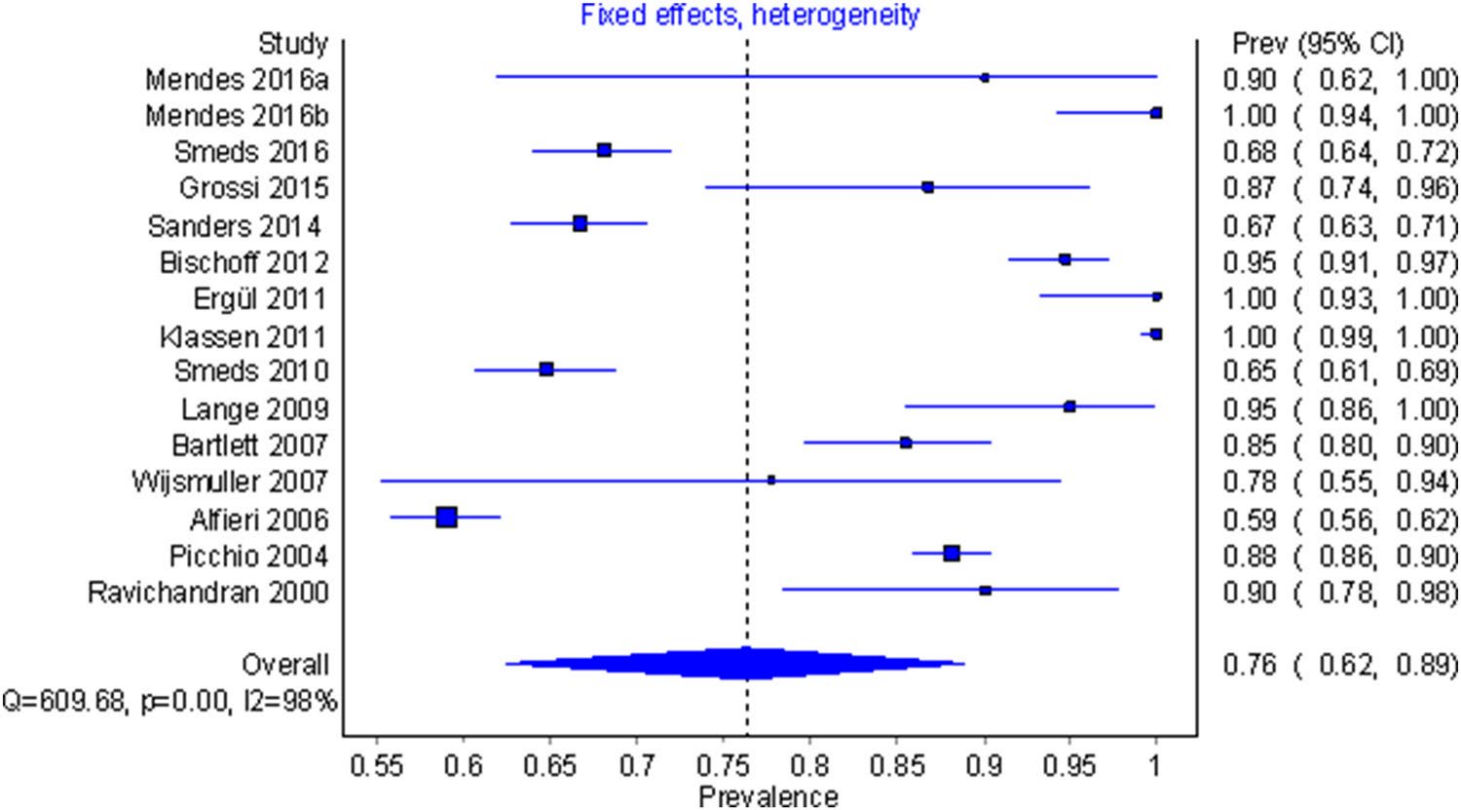
Pooled prevalence estimates (identification rates) of the IHN under a fixed-effects model with heterogeneity correction

**Table 5:** Pooled prevalence estimates (identification rates) of the IHN

Study group	*N*	Half-bodies	PPE% (95% CI) Random	PPE% (95% CI) Fixed	*I*^2^ (95% CI)	*Q*
All studies	15	4187	86.7 (78.3–93.3)	76.3 (62.5–88.9)	97.7 (97.1–98.2)	609.68^***^
Type of dissection						
Cadaveric	3	228	91.8 (66.8–1.00)	99.2 (74.5–1.00)	89.3 (70.9–96.1)	18.68^***^
During hernioplasty	12	3959	84.8 (76.5–91.6)	74.2 (61.5–86.0)	97.4 (96.5–98.0)	420.08^***^
Study center						
Monocenter	12	2154	91.4 (83.2–97.2)	86.6 (72.2–98.1)	96.0 (94.5–97.2)	227.66^***^
Multicenter	3	2033	64.5 (58.5–70.4)	63.4 (57.1–69.5)	87.1 (63.1–95.5)	15.45^***^
Geographic region						
Asia^a^	–	–	–	–	–	–
Africa^a^	–	–	–	–	–	–
Europe	11	3910	82.3 (73.3–89.8)	73.8 (61.2–85.5)	97.5 (96.6–98.1)	393.48^***^
North America	1	200	99.9^b^ (99.1–100.0)	99.9^b^ (99.1–100.0)	NC	NC
South America	3	77	92.6 (79.7–100.0)	93.3 (79.3–100.0)	67.8 (0.00–90.7)	6.22^*^

*PPE* pooled prevalence estimate, *NC* not computable because there was only one study in this group

*p < .05, **p < .01, ***p < .001

^a^There were no studies of IHN prevalence from Asia or Africa

^b^Fixed- and random-effects estimates are identical because the study-level prevalence rates were all 100.0% and there were was only one study

### Meta-analysis on the identification rate of the genital branch of the genitofemoral nerve

**Fig. 8:**
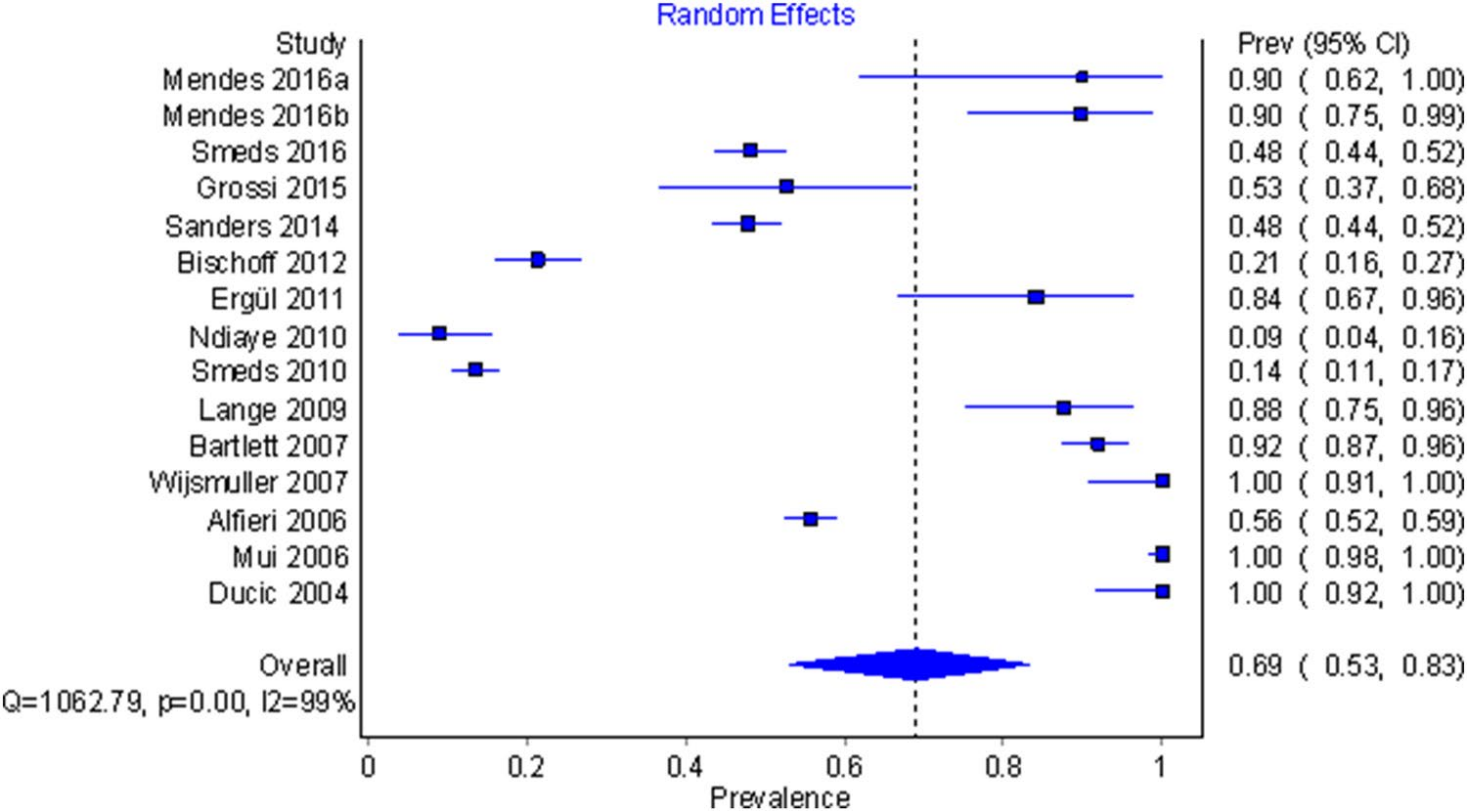
Pooled prevalence estimates (identification rates) of the GNF under a random-effects model

**Fig. 9:**
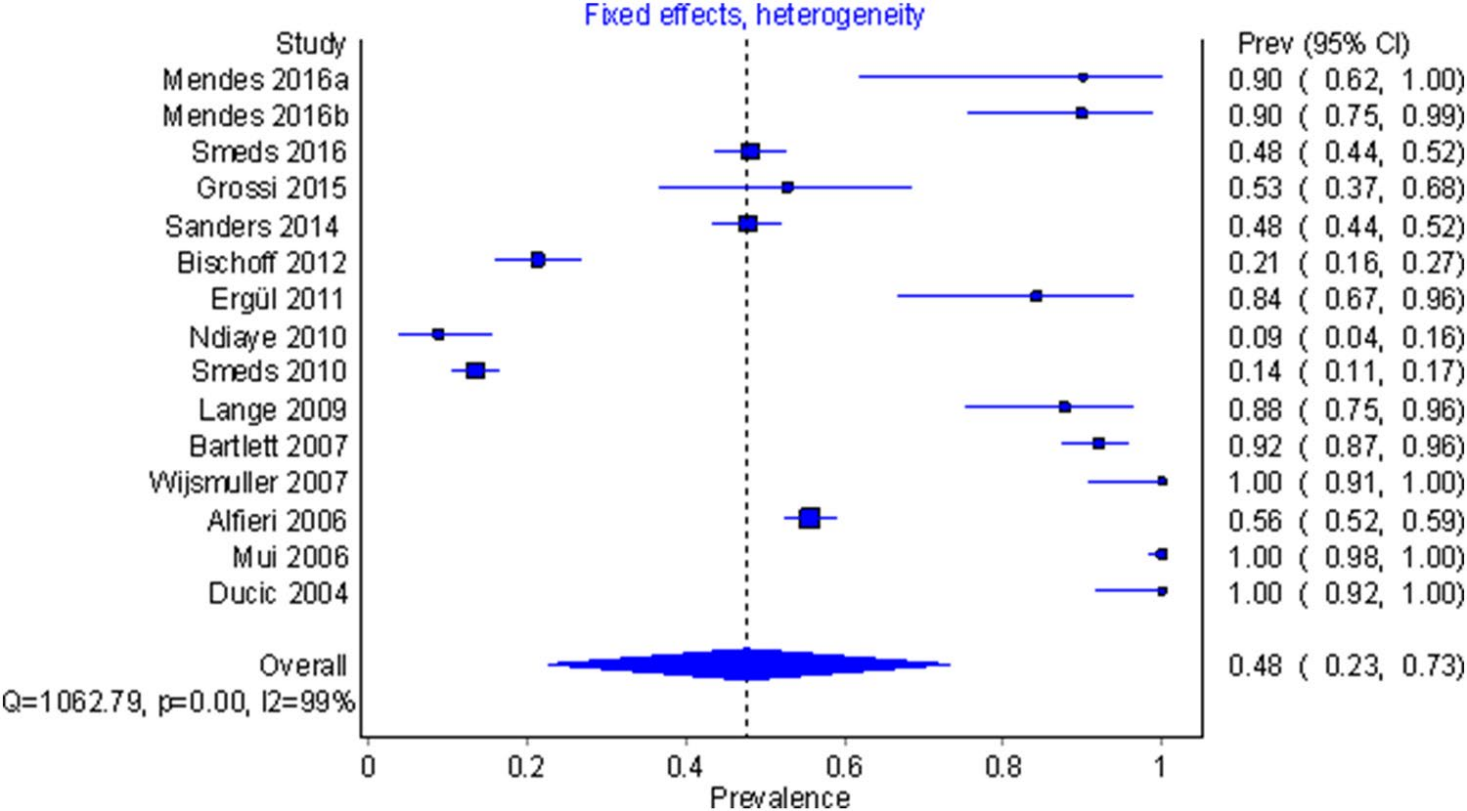
Pooled prevalence estimates (identification rates) of the GNF under a fixed-effects model with heterogeneity correction

Figures 8 and 9 and Table 6 show the results for the GNF. Fifteen studies and 3354 half-bodies were included. The identification rates for random-effects and fixed-effects models were 69.1% (95% CI 53.1%–83.0%) and 47.8% (95% CI 22.8%–73.0%), respectively. A leave-one-out sensitivity results ranged from 64.8 to 73.6% for a random-effects model and from 44.6 to 54.9% for a fixed-effects model. There was a large and statistically significant amount of heterogeneity overall and within subgroups. As with other outcomes, a visual analysis of funnel and DOI plots indicated irregularity and provided evidence that larger studies tended to have smaller prevalence.

**Table 6:** Pooled prevalence estimates (identification rates) of the genital branch of the genitofemoral nerve

Study group	*N*	Half-bodies	PPE% (95% CI) Random	PPE% (95% CI) Fixed	*I*^2^ (95% CI)	*Q*
All studies	15	3354	69.1 (53.1–83.0)	47.8 (22.8–73.0)	98.7 (98.4–98.9)	1062.79^***^
Type of dissection						
Cadaveric	4	148	79.6 (0.0–100.0)	38.7 (0.0–100.0)	98.1 (96.9–98.9)	160.20^***^
During hernioplasty	11	3206	65.6 (47.8–81.5)	48.2 (23.4–73.2)	98.9 (98.6–99.1)	897.33^***^
Study center						
Single center	12	1321	75.6 (46.1–96.7)	41.9 (0.0–90.4)	98.9 (98.7–99.1)	1020.63^***^
Multicenter	2	2033	50.7 (45.2–56.1)	51.6 (45.9–57.2)	NC	11.92^**^
Geographic region						
Asia	1	100	99.8^a^ (98.3–1.00.0)	99.8^a^ (98.3–1.00.0)	NC	NC
Africa^b^	–	–	–	–	–	–
Europe	10	3157	56.3 (39.0–73.0)	44.3 (21.5–67.7)	98.8 (98.4–99.1)	743.37^***^
North America	1	20	98.8^a^ (91.5–100.0)	98.8^a^ (91.5–100.0)	NC	NC
South America	3	77	79.0 (48.0–99.0)	72.7 (40.6–98.2)	84.5 (53.8–94.8)	12.91^**^

*PPE* pooled prevalence estimate, *NC* not computable because there were two or fewer studies in this group

*p < .05, **p < .01, ***p < .001

^a^Fixed- and random-effects estimates are identical because the study-level prevalences were all 100.0% and there were was only one study

^b^There were no studies of IHN prevalence from Africa

### Meta-analysis of anatomical reference points

Table 7 presents all secondary endpoints including the pooled estimates of distance of the point of the nerve emergence in relationship to the anatomic landmarks for the IIN, which was located inferior to the ASIS, medially to the ASIS, and the inguinal ligament. In one study [33], the range was reported instead of the standard deviation. Therefore, we estimated the standard deviation from the range using the guidelines in Hozo et al. [12] assuming an underlying normal distribution of nerve lengths. The distance from IIN emergence inferior to the ASIS was 2.8 cm (2.65–2.95) and medially to the ASIS was 3.62 cm (3.04–4.19). For nerve length outcomes, there was a large and statistically significant amount of heterogeneity. When computable, the heterogeneity estimates for other reference points were also large and statistically significant.

**Table 7:** Anatomical reference points for the ilioinguinal nerve

Reference point	Studies	*N*	PME (95% CI)	*I*^2^ (95% CI)	*Q*
Distance from the IIN emerged to					
Inferior to the anterior superior iliac spine	1	200	2.8 cm (2.65–2.95)	NC	NC
Medially to the anterior superior iliac spine	4	428	3.62 cm (3.04–4.19)	92.8 (84.9–96.6)	41.83***
Variations in the emergence of the nerve					
Posterior to the inguinal ligament	2	140	19.6% (12.7–27.5)	NC	1.18
Posterior to the anterior superior iliac spine	2	140	4.5% (1.0–9.8)	NC	1.37
Aberrant origin of the IIN from the genital branch of GNF	2	130	2.5% (0.4–6.0)	NC	0.95
Common trunk with the IHN	5	365	10.0% (2.0–23.3)	89.2 (77.5–94.8)	36.99***
Course of the IIN with regard to the spermatic cord					
Parallel	4	188	87.8% (46.6–100.0)	96.9 (94.5–98.3)	97.31***
Ventrally	4	188	57.2% (3.1–100.0)	97.6 (96.0–98.6)	126.97***
Type of exit of IIN from inguinal canal					
IIN exit through SIR	5	276	64.5% (19.0–99.0)	97.7 (96.3–98.5)	170.86***
Acute infero-lateral angulation of the IIN in close contact with and parallel to the SIR fibers at exit	3	168	4.9% (0.0–20.5)	89.7 (72.2–96.2)	19.36***
A plane superficial to the EOA having pierced it proximal to the SIR	5	276	14.6% (7.0–24.1)	70.5 (24.8–88.4)	13.55***
Mode of termination and branches					
Unique trunks					
Scrotal termination	1	110	36.4% (27.6–45.6)	NC	NC
Pubic termination	1	110	3.6% (0.8–8.1)	NC	NC
Femoral termination	1	110	2.7% (0.3–6.8)	NC	NC
Two branches	–	–	–	–	–
Three branches	–	–	–	–	–
Four branches	–	–	–	–	–

*PME* pooled mean estimate, *NC* not computable because there were two or fewer studies in this group, *SIR* superficial inguinal ring, *EOA* external oblique aponeurosis, *IIN* ilioinguinal nerve, *IHN* iliohypogastric nerve, *GNF* genital branch of the genitofemoral nerve

A random-effects model was used for all outcomes

*p < .05, **p < .01, ***p < .001

## Discussion

Inguinal hernia repair is one of the most commonly performed surgical procedures. Nowadays, the most frequent hernia repair is in the outpatient setting, which requires the use of local anesthesia, and the most frequent postoperative complication is late pain in the inguinal region [10]. Failure to identify inguinal nerves during the surgery has been correlated with the higher incidence of postoperative pain [7].

In this review we included 26 studies with 5265 half-bodies examinations. Fourteen studies were performed during inguinal hernioplasty and 12 during cadaveric dissections. We analyzed the identification rates of the nerves at the inguinal canal. The IIN nerve-identification rate was evaluated in 20 studies and its presence was reported in the 84.6% of the dissections. The IHN identification rate was evaluated in 14 studies and its presence was reported in 74.2% of the half-bodies’ examinations. The identification rate of the GNF was evaluated in 14 studies and the presence of nerve was reported in 47.34% of the cases. The analysis of the identification rates show that it is not always possible to correctly locate all of inguinal nerves and that the nerve that is most difficult to locate is the GNF. Identification rates obtained in this study were lower than the rates reported by a narrative review performed on 13 studies: 96% for IIN, 94% for IHN and 90% for GNF [1]. In addition, the identification rate was higher in cadaveric studies (identification rate for IIN: 97.27%, for IHN: 97.8%, for GNF: 37.83%) than in inguinal hernioplasty studies (identification rate for IHN: 63.52%, for IIN: 82.43%, for GNF: 47.8%) for all the nerves. This suggests in both cases the difficulty of identification of the GNF and that the different techniques used in anatomical and surgical procedures provide different outcomes.

Moreover, the identification rates of nerves varied across different geographic regions. There was a relatively higher identification rate of nerves reported in Asian studies and North American studies. In South America, the identification rate was very high for GNF. The data were very heterogeneous in other regions. In Africa, there was a identification rate of zero for IHN and GNF. In Europe, the identification rate was 4.4% for GNF, 72.35% for IHN, and 81.9% for IIN. We suspect the heterogeneity of patients and settings may have resulted in much of the heterogeneity between studies.

Finally, we also found that the study size was a predictor of the identification rate. Larger studies tended to have lower identification rates. Large sample size study being correlated with outcomes is a phenomenon that Sterne et al. [13] hypothesize could be the result of “interventions being implemented less thoroughly in larger studies, resulting in smaller effect estimates compared with smaller studies”. The outlier study by Lange et al. [25] with a low nerve identification rate may be explained by the small sample size of patients included (*n* = 40). However, in this study, the authors used methods to increase reliability, with each identified nerve being photographed by the operating theatre nurse as proof which was rechecked by the surgeon and then reviewed by an anatomist.

In modern abdominal wall surgery, inguinal nerve identification plays an increasingly important role and represents a source of significant benefits. Nonetheless, inguinal nerve variants have always been a pitfall for surgeons and the fact that all structures cannot be located in all cases, as also this review demonstrated, has important repercussions for surgical practice. The European Hernia Society guidelines [10] recommend the identification of the three inguinal nerves (ilioinguinal, iliohypogastric and genital branch of the genitofemoral) for the reduction of late postoperative pain deriving from nerve injuries.

The first description of abdominal pain after inguinal surgery was reported as “genitofemoral causalgia” from Magee in 1942 [37]. Heise and Starling [38] described the chronic pain after hernioplasty treated with partial or total prosthesis removal as “mesh inguinodynia”. There are many controversies about the treatment of the identified inguinal nerves: Lichtenstein et al. [39] for example, proposed the preservation of the inguinal nerves after identification; other surgeons suggest the prophylactic neurectomy [40]. However, there is no evidence of the superiority of one of the two techniques in postoperative pain reduction.

Surgeons who mainly perform hernioplasty surgery have the best outcomes in terms of identifying nerve structures [41]. The success in surgical identification of the three nerves has been found to be largely associated with surgical skills [41], but sometimes some anatomical variations of the nerve topography makes the surgical identification difficult no matter the skill level, especially in the cases were some of inguinal nerves are not present. For these reasons, standardization of education and training in nerve identification in hernia surgery is needed [41, 42].

Our meta-analysis reported statistical analysis of nerve course variations to provide more reliable points of reference for safe and correct local anesthesia, that would allow lowering of the incidence of chronic postoperative inguinal pain. For most nerve length outcomes, there was a large and statistically significant amount of heterogeneity. The data on anatomical reference points were in contrast with the data reported in classic anatomical textbooks, such as Clinical Anatomy by Regions [43]. Those authors suggest performing an anesthetic block of IIN and IHN 2.5 cm above the anterior superior iliac spine on the spinoumbilical line [43]. To ensure proper identification of inguinal nerves, ultrasonographic confirmation of their location should be attempted [4, 9]. In cases of abnormal nerve courses, the successful application of blind anesthetic blocks may be impossible.

## Conclusion

Our systematic review and meta-analysis provided the largest and most comprehensive up-to-date data on the identification rates of the inguinal nerves. The identification rates of the inguinal nerves in our study was lower than reported in literature. The lowest was found for the genital branch of genitofemoral nerve suggesting this nerve was the most difficult to identify. Moreover, the nerve topography results must be taken in account in the nerve sparing approach during hernioplasty. The knowledge about anatomy of inguinal nerves can facilitate their proper identification and reduce the risk of iatrogenic injury and postoperative pain.
